# The one health perspective to improve environmental surveillance of zoonotic viruses: lessons from COVID-19 and outlook beyond

**DOI:** 10.1038/s43705-022-00191-8

**Published:** 2022-10-30

**Authors:** Mats Leifels, Omar Khalilur Rahman, I-Ching Sam, Dan Cheng, Feng Jun Desmond Chua, Dhiraj Nainani, Se Yeon Kim, Wei Jie Ng, Wee Chiew Kwok, Kwanrawee Sirikanchana, Stefan Wuertz, Janelle Thompson, Yoke Fun Chan

**Affiliations:** 1 Singapore Centre for Environmental Life Sciences Engineering, Nanyang Technological University, Singapore, Singapore; 2 Department of Medical Microbiology, Faculty of Medicine, Universiti Malaya, Kuala Lumpur, Malaysia; 3 Department of Medical Microbiology, University Malaya Medical Centre, Kuala Lumpur, Malaysia; 4 Research Laboratory of Biotechnology, Chulabhorn Research Institute, Bangkok, Thailand; 5 Centre of Excellence on Environmental Health and Toxicology, CHE, Ministry of Education, Bangkok, Thailand; 6 School of Civil and Environmental Engineering, Nanyang Technological University, Singapore, Singapore; 7 Campus for Research Excellence and Technological Enterprise (CREATE), Singapore, Singapore; 8 Asian School of the Environment, Nanyang Technological University, Singapore, Singapore

**Keywords:** Biomarkers, Environmental sciences, Infectious diseases, Molecular biology

## Abstract

The human population has doubled in the last 50 years from about 3.7 billion to approximately 7.8 billion. With this rapid expansion, more people live in close contact with wildlife, livestock, and pets, which in turn creates increasing opportunities for zoonotic diseases to pass between animals and people. At present an estimated 75% of all emerging virus-associated infectious diseases possess a zoonotic origin, and outbreaks of Zika, Ebola and COVID-19 in the past decade showed their huge disruptive potential on the global economy. Here, we describe how One Health inspired environmental surveillance campaigns have emerged as the preferred tools to monitor human-adjacent environments for known and yet to be discovered infectious diseases, and how they can complement classical clinical diagnostics. We highlight the importance of environmental factors concerning interactions between animals, pathogens and/or humans that drive the emergence of zoonoses, and the methodologies currently proposed to monitor them—the surveillance of wastewater, for example, was identified as one of the main tools to assess the spread of SARS-CoV-2 by public health professionals and policy makers during the COVID-19 pandemic. One-Health driven approaches that facilitate surveillance, thus harbour the potential of preparing humanity for future pandemics caused by aetiological agents with environmental reservoirs. Via the example of COVID-19 and other viral diseases, we propose that wastewater surveillance is a useful complement to clinical diagnosis as it is centralized, robust, cost-effective, and relatively easy to implement.

## Introduction

### Emerging infectious diseases and the one health approach

Viral pathogens have been identified as causing pandemics in human populations as long as 12,000 years ago, when previously nomadic humans settled into villages and domesticated animals [1]. More recently, ever-increasing proximity between humans and wildlife due to population dynamics, as well as industrial livestock practices, have further escalated the likelihood of encountering potentially pandemic zoonotic viruses that are able to ‘jump’ the species barrier [2].

**Fig. 1:**
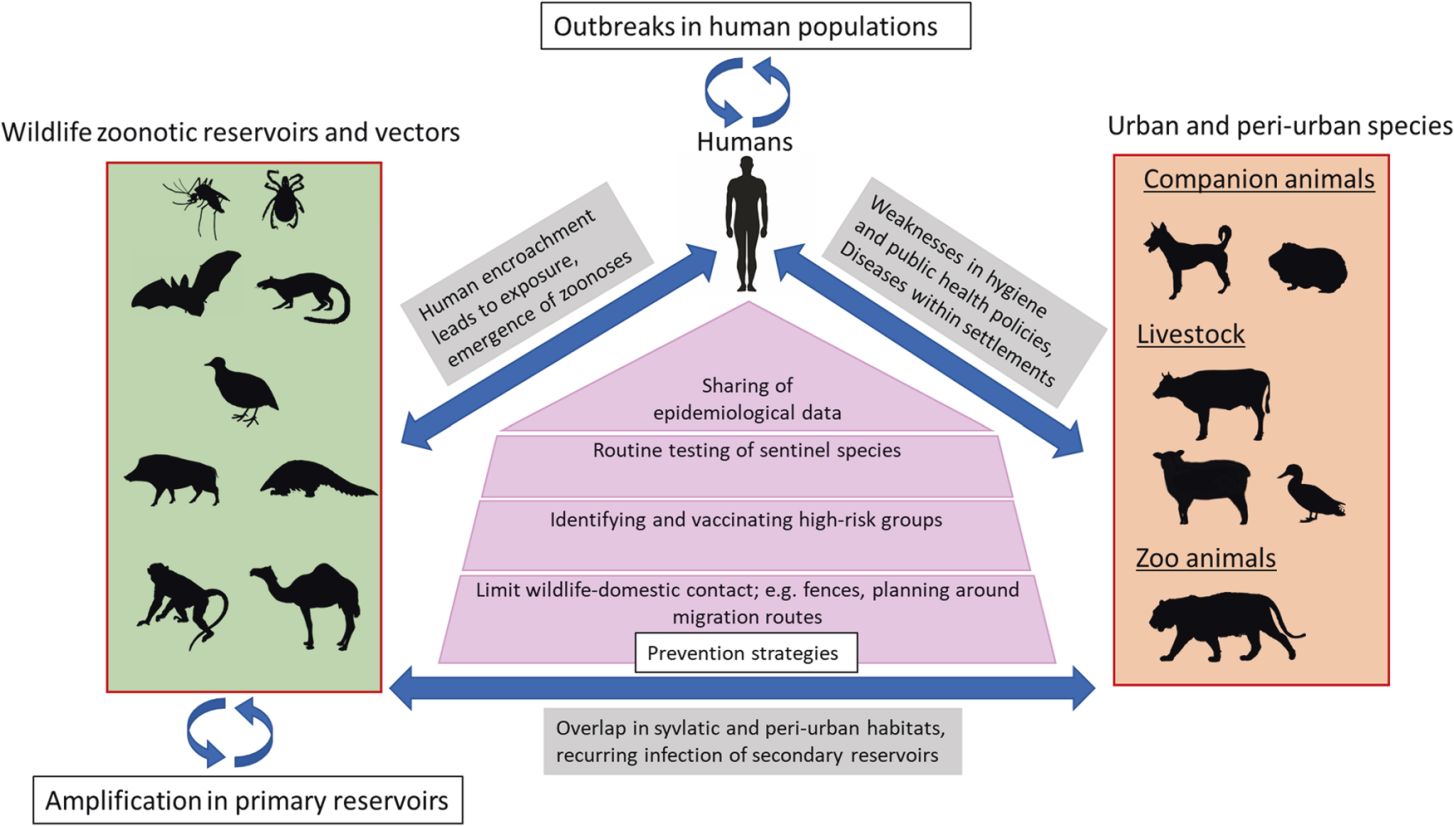
Schematic overview of the circular interaction between wildlife (green box) and urban (orange box) zoonotic infection reservoirs and the human population. Overview of zoonotic infection pathways between domesticated and non-domesticated (i.e., wildlife) animals and humans (based on Lazarus, Fosgate [91]).

Emerging infectious diseases (EID) are infections whose transmission range has rapidly expanded to previously naïve populations. An estimated 75% of all EIDs have a zoonotic origin and circulate relatively safely in their animal hosts. Zoonoses breech the species barrier in environments where a human-animal interface is commonplace but are invariably shown to be exacerbated by anthropogenic ecological disturbances, such as urbanisation and climate change. One Health aims at merging scientific insights from disciplines concerning animal, human, and environmental health to reduce the overall disease burden (Fig. 1). The One Health approach was first formalized by the formation of the One Health Initiative Task Force in 2007 [3] and the One Health Commission in 2008 [4].

An important application of One Health is the quantitative and qualitative analysis of viruses in the aquatic environment, which has been long established for the purpose of identifying diffuse pollution sources (e.g., from untreated sewage) in water quality monitoring [5]. While culture-based methods like the cultivation of faecal indicator bacteria have been employed for almost a century now, assays relying on more sensitive molecular detection and quantification have become more common. Occurrences and outbreaks of enteric RNA viruses such as norovirus and rotavirus, for example, have been well documented to occur in recreational settings such as freshwater lakes and beaches [6], as well as in food items irrigated with insufficiently treated sewage [7].

Consequently, the assessment of viruses in aquatic environments (e.g., rivers and lakes) has become more relevant due to the wider availability of molecular detection capabilities like quantitative (qPCR) and digital polymerase chain reaction (dPCR). Molecular assays based on qPCR and dPCR are capable of targeting enteric (i.e., human pathogenic) viruses frequently shed by symptomatic and asymptomatic individuals have been successfully used in a plethora of case studies worldwide [8]. More recently, they have also been included into standardized methods, such as in recommendations proposed by the International Organization for Standardization [9], the World Health Organization (WHO) [10] and the World Bank [11]. Human-adjacent pathogens such as the plant-based pepper mild mottle virus have also been measured through wastewater surveillance, in an effort to understand how the aquatic environment (and by extension aquatic and marine wildlife) are exposed to anthropogenic influences [12].

Seeing environmental surveillance in light of the integrated concept of One Health (e.g., by utilizing assays, experiences, and knowledge of the occurrence and abundance of human, plant, and animal viruses) can provide valuable tool sets in combatting preventable emerging diseases, as well as minimize the risk of human and viral outbreaks. Here we highlight examples of sources and factors aiding the spread of zoonotic infection and discuss how the understanding of modes of transmission can help with disease surveillance.

## “Disease X” and (zoonotic) emerging virus infectious diseases

### Categorization, relevance, and burden of disease of EID

The WHO [13] proposed seven virus-associated infections as most urgently needing research and development preparedness: (1) Crimean Congo haemorrhagic fever; (2) filovirus diseases (i.e., Ebola virus disease and Marburg virus disease); (3) Highly pathogenic emerging coronaviruses (CoV) relevant to humans (Middle East Respiratory Syndrome (MERS) CoV & Severe Acute Respiratory Syndrome (SARS) CoV); (4) Lassa fever; (5) Nipah; (6) Rift Valley fever, and (7) “Disease X”, a yet unknown or novel pathogen, most likely of zoonotic origin and capable of infecting the respiratory tract in humans and/or animals (see Table 1). While not without controversy, the putative “Disease X” has been proposed by the WHO as a placeholder for the unknown pathogen in 2018, to raise awareness and facilitate prospective research and infection control and prevention capabilities [14].

**Table 1:** Most relevant virus-associated disease according to the corresponding pathogen, animal vector, annual case load (and fatality rate), global distribution and availability of treatment.

Disease	Associated pathogen	Detectable in the environment	Animal vector	Number of annual cases / Epidemiological relevance	Area	Treatment available	Reference
Crimean–Congo haemorrhagic fever (CCHF)	CCHF virus	Yes (shed in stool)	Tick bites or close livestock contact	Frequent outbreaks with 10–40% fatality rate	Europe (Balkans, Turkey), Asia, Middle East, and Africa	No	[109]
Filovirus disease	Filoviruses (particularly Ebola virus and Marburg virus)	Yes (e.g., Ebola virus is shed in stool and urine)	Primates, pigs, and bats	Frequent outbreaks with average 50% (up to 90% in the past) fatality rate for Ebola Virus, and up to 88% for Marburg Virus	Sub-Sahara Africa (Uganda, DR Congo, Kenya, and Angola), South America (Brazil)	Prevention via vaccination and treatment via approved monoclonal antibody treatment (Ebola)	[110]
Emerging coronavirus diseases (COVID)	SARS-CoV, MERS-CoV, SARS-CoV-2	Yes (shed in stool)	Bats and camels	607 million cases, 6.50 million fatalities for COVID-19 as of September 15^th^, 2022	Worldwide	Prevention via vaccination and treatment via licensed antivirals for SARS-CoV-2	[111]
Lassa fever	Lassa virus	Yes (shed in urine and stool)	Rat and mouse faeces	300,000–3,000,000 cases, 5,000 deaths	Western Africa (Guinea, Nigeria, Sierra Leone, and Liberia)	limited (experimental usage of ribavirin)	[112]
Nipah virus infection	Nipah virus	Yes (shed in stool and urine)	Fruit bats	Frequent outbreaks with 40–75% fatality rate	Malaysia, Singapore, Bangladesh, and India	Under development	[113]
Rift Valley fever	Rift Valley fever Virus	No (no shedding in urine or faeces in any species known)	Mosquitoes	Transmissions to humans are suspected but not confirmed	Sub-Sahara Africa	No	[114]

Outbreaks of EIDs inflict considerable costs to public health and the economy of populations. Burdens on patients may range from high mortality rates (Ebola [15] and congenital Zika [16]), to causing chronic debilitating conditions in survivors (such as chronic inflammation post-chikungunya infection [17]). Besides economic costs in treating these infections, countries may additionally invest in surveillance and prevention strategies (e.g., vector control and drug development [18]). Disease linked to livestock (e.g., avian influenza [19]) has implications for food production and supply industry [20]. Diseases with reservoirs in wildlife and the environment (e.g., Nipah [21]) can involve seasonal outbreaks. Zoonotic diseases with multiple reservoirs and vectors (e.g., tick-borne encephalitis virus [22]) can have unpredictable and sporadic outbreaks. The frequency and unpredictability of outbreaks may limit the efficacy of management and elimination strategies where surveillance has limited range [23], especially in regions where human-wildlife conflicts are increasing due to encroachment into wildlife refuges [24].

### SARS-CoV-2 and the case for wastewater surveillance

The coronavirus disease 2019 (COVID-19) and its aetiological agent, the severe acute respiratory syndrome coronavirus 2 (SARS-CoV-2), fits well into the criteria of a putative “Disease X” as formulated by the WHO. Since its onset in 2020, the COVID-19 pandemic has revealed that even the most highly developed and (at least theoretically) best prepared healthcare systems worldwide struggle when encountering a previously unknown viral infectious disease of zoonotic origin.

In the ongoing COVID-19 pandemic, environmental surveillance of wastewater (WWS) has been shown to be a uniquely valuable tool to determine the emergence of local clusters and transmission trends in defined sewersheds. The suitability of environmental surveillance for viruses such as SARS-CoV-2 have resulted in their addition in guidelines by the WHO [10], the World Bank [11], the European Commission [25] and the US-CDC [26]. WWS campaigns implemented worldwide also possess the potential to generate a treasure-trove of spatiotemporal data that is currently being used to generate an international “wastewater virome” [27]. Insight into the entirety of all (animal, human and plant) viruses in this environment will not only help establish sampling and quality control protocols, but also enable researchers to be prepared to respond quickly for environmental surveillance of novel and emerging, or variants of already known human-pathogenic viruses.

While the molecular assays currently used in the context of environmental virus surveillance lack the ability to indicate the target’s ability to cause infection, monitoring the occurrence of SARS-CoV-2 in wastewater in the community has already been proven to reveal unique insights into local and overall pandemic trends [28]. At the building level, wastewater surveillance with its fast turn-around times, can identify local outbreaks and allows for the implementation of non-pharmaceutical intervention or testing campaigns to reduce the formation of clusters [29]. Furthermore, active- or passive monitoring campaigns of airborne SARS-CoV-2 in indoor settings showcases the possibilities of using this method to survey virus loads in public spaces like schools, hospitals, public transportation, or highly frequented workplaces like offices [30].

**Fig. 2:**
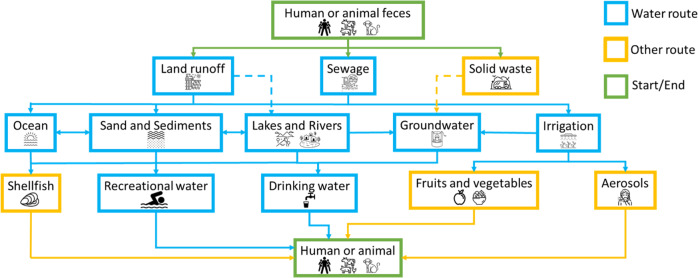
Schematic overview of fecal contamination routes in the aquatic environment. Blue boxes and lines relate to waterborne contamination, yellow to water indirectly water associated routes and green boxes depict the start / end point of the contamination. Contamination routes for waterborne pathogen of human and animal faecal origin (based on Rodríguez-Lázaro, Cook [31]; dotted line indicates scientific dispute in literature). Among the points between the excretion and uptake of aetiologic agents at which environmental surveillance has been employed or proposed are sewage (sampled either at manholes or centralized in WWTP), marine, freshwater and groundwater, shellfish and other food items which could have come into contact by greywater irrigation.

While surveillance efforts for SARS-CoV-2 are currently in the limelight, more than 150 enteric viruses are currently known to be relevant to human and animal health and are associated with various environmental transmission routes, each of which could be considered for surveillance to break potential infection cycles (see Fig. 2). While certain transmission paths are undisputed and relatively easy to monitor (highly persistent viruses remain infectious and detectable during all seasons and after wastewater treatment), others are not yet exhaustively investigated [31].

Besides the more widespread applications in the current pandemic, the environmental monitoring of vector-borne viruses is a prime example of the feasibility of applying the One Health concept in a public health context [32]. Arthropod borne viruses (arboviruses) like dengue- and Zika virus, are transmitted via mosquito or ticks and are known to manifest in a range from asymptomatic to symptoms that can be easily mistaken for mild fevers or cold. As they are predominantly found in parts of the world where clinical infrastructure and sophisticated diagnostic capabilities can be limited, environmental surveillance offers a unique alternative to ensure high-resolution diagnostics for their occurrence [33].

## From animals and pathogens: what drives the zoonotic potential?

### Common zoonotic pathogens

Environmental surveillance, informed by host organism ecology, can help track zoonotic pathogens across multiple stages of their transmission cycles. Understanding the virus-host interaction is vital to predicting where and how cross-species transmission (“spill-over”) events from one species to another can occur, as viruses are incapable of replication and thus reliant on a host’s cell machinery to reproduce. For example, RNA viruses such as dengue virus [34, 35], and the human immunodeficiency virus (HIV) [36] have been reported to be considerably more likely to infect a host outside their natural range than DNA viruses (e.g., herpes B virus [37]) due to the lack of proofreading mechanisms. Unlike this extra fail-safe in DNA viruses, virions with an RNA genome are significantly more likely to accumulate mutations beneficial for adapting to new hosts during amplification [38]. Virus capability to replicate in the host cytoplasm also aids in adaptability over the need to enter the host nucleus [39].

Compilation of data on host-virus interactions suggests that rodents, bats, and primates are major reservoirs of zoonotic pathogens as they collectively harbour around 75% of described zoonotic viruses [40, 41] (see Fig. 1). Traits such as having fast-paced metabolisms, as well as relatively lower life expectancies and shorter reproduction cycles, are also considered to positively factor into the efficacy of rodents and bats as disease spreaders [42]. Furthermore, animals with higher reproduction rates (i.e., r-selective) that produce larger numbers of immunologically naïve offspring have been reported to be even more susceptible to infections with RNA viruses capable of cross-species transmissibility [43]. It has been predicted that organisms with ‘fast’ life histories invest more in nonspecific and inflammatory immune defences at the cost of adaptive immunity [44, 45]. Fast-lived species also tend to be habitat generalists, able to match their high reproductive rates with great dispersal ability into novel environments [46], putting them into close proximity with others. This context helps to prioritize environmental surveillance efforts to target these more likely reservoirs for zoonosis emergence.

### Geographical restriction and global hotspots

Many important zoonotic diseases can be traced back to tropical regions of the world that contain high biodiversity while also being synonymous with pervasive land-use changes [47], ecotourism and animal exportation [48]. Examples for this are Ebola [49] and West Nile virus [50] in Africa, as well as Nipah [51] and MERS [52] in Asia.

Biodiversity loss can have contrasting effects on disease persistence, as declines in host populations attenuates the transmission cycle for diseases requiring vectors, or those that are highly specific [53]. However, parallel declining numbers in a predator population allows for some reservoir hosts to proliferate better [54]. Therefore, high biodiversity reduces zoonotic spill over risk through dilution effects; vectors would have greater prey choice in a pristine versus degraded forest, from which some prey would be poor hosts and result in ‘wasted bites’ [55]. Ostfeld [56], for example, highlighted a dilution effect on mosquito-borne West Nile Virus disease in regions threatened with biodiversity loss, thus suggesting that human cases arose due to declines in preferred hosts (e.g., small robins).

### Similarities in ancestry and sympatry

Pathogens which are most likely to cross to humans are usually those that require fewer mutations to bypass genetic barriers and infect human cells [57]. This hypothesis would predict an increased risk of host shifts from pathogens of closely related non-human primates [58], and low risk of zoonoses from more distantly related taxa [59]. The simian immunodeficiency virus (SIV) group which includes precursors for HIV, for example, is carried by many primate species [60]. Zoonoses are therefore more likely to occur in hosts that are sympatric with human communities, which includes domesticated (i.e., raised and kept for human benefit such as dogs, hamsters, and cats) and synanthropic species (i.e., wildlife that live around human settlements such as rats and racoons) (Fig. 1) [61]. Morand, McIntyre [62] have found that species with the longest history of domestication such as dogs and ungulates had the highest number of shared pathogens with humans. Several temperate diseases have possible origins in domesticated animals such as ruminants and have been beneficial in the discovery and development of the first vaccines (e.g., measles [63], smallpox [64]). Sites where there are close interactions between humans and the animal kingdom have been proposed as prime locations for environmental surveillance efforts, even before the onset of the current COVID-19 pandemic [65].

### Animal behaviours and traits

Foraging behaviours are a key source of cross-species transmission of disease between animal species (as well as humans). Carnivores are hypothesised to accumulate pathogens through their prey, and hence have a parasite richness that correlates to the diversity of prey in their respective diets [66]. Consequences of frequent exposure to pathogens include immunological adaptations such as greater white blood cell count in top predators [67]. The breadth of the host’s diet is further positively correlated to their accumulation of microparasites due to diverse exposure [68]. Similarly, vectors of zoonotic disease tend to feed on multiple host species, allowing populations to adapt to varying host availability [69]. For a vector-borne pathogen to cause zoonotic concern, it’s vector typically would preferentially feed on humans [70]. Seasonal migration (e.g., birds and bats) has implications for vectoring disease across routes and creating loci of endemism for pathogens outside of usual home ranges of hosts [71]. Although migration is not shown to predict high zoonotic virus numbers, the physiological stress experienced by migrating animals can cause immunosuppression and increase their susceptibility to acquiring disease or re-emergence of latent infection [72].

### From humans and the environment: what drives the zoonotic potential and how to utilize it for surveillance

Other than the acquisition of viral respiratory, vector borne, or gastrointestinal pathogens, the spread of infectious disease between species is a multistage process that is strongly influenced by human and environmental factors. Drivers of transmission have been observed to act on reservoirs and vectors to increase transmission, prevalence, and establishment of disease in a population [57]. While potentially causing an additional burden of disease by facilitating a more widespread transmission of zoonotic infections, the same drivers can also be utilized to optimize environmental surveillance strategies.

### Climate change and human behaviour

As broad-scale environmental change occurs (e.g., climate change and landscape modification), the distribution of species can also change, favouring species implicated in disease transmission and driving disease emergence [73]. Climate factors such as temperature and humidity are shown to be highly correlated with mosquito populations [74, 75]. While frequent rainfall creates more outdoor bodies of water for mosquitoes to breed [76], periods of drought lead to more water storage structures in human settlements, thereby also increasing the number of viable breeding sites [77]. Utilizing the insights, processes, and knowledge gained during COVID-19 surveillance campaigns, the concerted samplings of stagnant waterbodies in informal settlements and storage containers in water scarce environments could easily be conceived to facilitate One Health inspired environmental surveillance [78].

Outbreaks of vector-borne disease can originate from infected vertebrate hosts that enter an immune-naïve population (e.g., human travellers and inter-continental livestock trade [52]). One of the best described examples of this phenomenon is the introduction of the Japanese encephalitis virus, which most likely spread from Asia to Australasia by the movement of reservoir hosts due to easier global mobility of humans and animals (e.g., pigs and birds for human consumption), thereby allowing for gradual transmission cycles to sustain infected populations across countries [79]. The expansion of industrial scale meat production and trade has further increased available hosts for diseases, particularly due to crowded livestock transportation (e.g., ships and trucks) and housing facilities that allow for a rapid spread and recombination of RNA viruses [80]. Another example of this is the spread of the African swine fever virus, which likely found its way into wild boar populations in Europe via hogs meant for consumption. The occurrence and transmission of this virus, which could show pandemic potential if large scale livestock production facilities get infected, is commonly detected by “One-Health” inspired sentinel testing of the local boar population [81].

## Preparative and proactive planning

### Targets and methods for surveillance and one health sentinels

Surveillance and mitigation of EID requires an interdisciplinary, comprehensive, robust, and data driven One Health approach. Combining expertise from the fields of public health, ecology, and urban planning [82], as well as clinical- and environmental virology, offers the most feasible route of achieving this [83]. Efficient monitoring of EIDs may include reactive approaches (following an outbreak) such as establishing quarantine zones for infected species [84], treating the disease where possible, and culling if necessary [85], as well as proactive measures like targeting the most vulnerable groups within a population (animal or human) by vaccination programs [25]. Passive broad-scale surveillance, and monitoring the distribution of identified reservoirs, have also been shown to be beneficial - not least during the COVID-19 pandemic as well as seasonal outbreaks of dengue [35, 86].

### Human-animal interface and data sharing bodies

Preventing disease emergence through the erection of physical barriers (e.g., building fences to control the spread of rabbit populations [87]) alone is ineffective for pathogens circulated through the environment [88], as well as migratory flying hosts [89]. In South Africa, for example, successful efforts to monitor and contain the spread of foot-and-mouth disease (as well as their aetiological enterovirus agent) included perimeter fencing around Kruger National Park to separate cattle from wild ungulates carrying the pathogen [90], as well as testing sentinel animals and subsequently vaccinating livestock and local wildlife against the disease [91, 92]. Transmissible vaccines are particularly effective for gregarious species or those that practice social allogrooming [93]. Low coverage vaccination strategies are sufficient to avoid large outbreaks of disease if high risk populations are selected for immunisation [94]. Urban enclosures that house collections of animals such as zoos and botanical gardens are also potential venues to set up One Health inspired environmental surveillance campaigns. Indeed, several studies have identified agricultural and human-health relevant viruses in Zoos (e.g., Schmallenberg virus and COVID-19, respectively) [1, 2] and sentinel species have been identified that may provide early warnings for emergence potentially zoonotic viruses (such as COVID-19 infected minks or felines) before human cases occur [95, 96]. Public health and epidemic-preparedness research bodies have called for a standardised format in collecting and disseminating information on past and current outbreaks to provide predictive power to anticipate future disease emergence. These include databases and tools for analysing host-pathogen interactions, reports on discovery and surveillance strategies [97], and discussions on policies and sampling campaigns [98].

During ongoing SARS-CoV-2 surveillance efforts, certain limitations of commonly used approaches and methods have emerged, including challenges associated with data sharing. Several initiatives including “COVIDPoops” [95, 99], and others in Europe [10, 30], have enabled sharing across international boundaries, which has assisted with dissemination of protocols and enabled some degree of standardization across research groups. Utilization of molecular surveillance data from wastewater requires in-depth knowledge of the catchment upstream of the sampling point (regardless of sampling the downstream treatment plant of individual manholes). Wu, Lee [100] and Lee, Gu [35] further defined that inherent detection and quantification limits of assays commonly used in WWS require minimum populations upstream of the sampling point. As these minima can range in the thousands, small or remote communities might encounter similar challenges as areas with a highly mobile population such as commonly found in informal settlements. The same assays are also prone to inhibition due to the complexity of the wastewater matrix, which requires constant attention of the laboratory personnel [101]. Therefore, the “data” that results from a WWS campaign to include in a standardized reporting framework includes not only measurements of the target of interest, but also site description, and assay validation information such as limit of detection and inhibition checks.

## Environmental surveillance via the One Health approach

### WWS can identify hotspots before susceptible groups are at risk

Depending on clinical testing capability and intensity, WWS can provide an early warning regarding pathogen circulation. For example, WWS is able to detect SARS-CoV-2 variant up to 14 days earlier than a clinical case illustrating its great potential can be overcome with improved laboratory protocols and better bioinformatics tools [28]. The expertise and infrastructure developed through the “lessons-learned” from the environmental surveillance of SARS-CoV-2 can help pave the development of similar efforts for zoonotic diseases that are already widespread or that harbour a pandemic risk. In countries and regions where, clinical diagnostic capabilities are underdeveloped, environmental surveillance (e.g., of communal wastewater), the monitoring of sentinel animal and particularly susceptible human populations can serve to a complement cost-and labour-intensive clinical approaches [102]. Arthropod-borne viruses like dengue virus and yellow fever virus are causing a significant burden of disease and annual economic losses in tropical regions worldwide are a prime target for such surveillance efforts, as traditional, monitoring focussing on clinical diagnostics is long known to be insufficient. Laborious (sylvatic) surveillance of mammalian hosts or insect vectors in a defined geographical context could act as a bridging technology until more systematic and tailored surveillance strategies of communal sewage are introduced [102, 103]. Moreover, comparable to SARS-CoV-2 monitoring efforts, the timely detection of arbovirus genomes in communal sewage could allow for the implementation of non-pharmaceutical interventions such as the removal of stagnant water from streets and parks as potential mosquito breeding grounds, or the distribution of mosquito nets or more drastically the release of genetically modified male mosquitoes [104]. More precise knowledge about the spatiotemporal occurrence of zoonotic viruses with high burden of disease such as arboviruses via wastewater surveillance would further allow for a better and more guided community engagement, which could in turn help build trust and reduce the breeding grounds for insect vectors [105].

Regular exchange of information and data through established collaborative networks could lead to the exchange and development of protocols and methodologies, such as those for the molecular SARS-CoV-2 variants of concern qPCR assays targeting clinically relevant genome regions that are not stable enough to be detected in the environment [100, 106] or genome motifs associated with previously unknown zoonotic viruses, whose occurrence in wastewater could help in identifying them before they are introduced into a larger human or animal population [10, 11].

## Conclusion

More and more molecular methods that enable researchers for the surveillance of viral pathogens with cross-species transmissibility in close to real time emerged in the past two years, as a result of the COVID-19 pandemic [107]. At the same time, the ease and availability of genome sequencing analysis for known genome targets, as well as gene motifs associated with yet unknown (zoonotic) viruses with pandemic potential (“Disease X”), will most likely greatly accelerate the ability of researchers worldwide to identify, monitor and consequently suppress the spread of virus associated diseases [14, 27].

Considering the likelihood of the (re-) emergence of the next zoonotic virus that is capable of causing a pandemic, it is imperative to explore cost- and labour efficient environmental surveillance methods in a standardized and universally applicable manner [100]. The first steps in this direction have been taken by the implementation of more and more standardized WWS infrastructures worldwide [99]. Unlike clinical diagnostics, One Health inspired environmental surveillance approaches are by design intended for the centralized monitoring of larger populations, as well as a longitudinal assessment of local outbreaks. The lower per-person cost that comes with such a centralized approach can thus enable public health practitioners in high and low resource environments to quickly identify the (re-) emergence of EID-associated outbreaks and local clusters and implement public health interventions [108].

The increasingly sophisticated methodologies that have emerged in the past two years also allow for the non-targeted analysis of wastewater, due to the availability of sequence analysis capabilities that can be used not only for known genome targets, but also those gene motifs associated with yet unknown (zoonotic) viruses that may end up being responsible for “Disease-X” [14, 27].

## Data Availability

The authors confirm that all materials, data, code, and associated protocols will be available to readers without undue qualifications and upon request.
